# Enhancing In Vitro Regeneration in Three Sweet Potato Genotypes: Interplay Between Disinfectant, Explant Age, and Genotype

**DOI:** 10.3390/biotech14030063

**Published:** 2025-08-19

**Authors:** El Hadj Hussein Tapily, Kan Modeste Kouassi, Marius Konan Kouassi, John Steven S. Seka, Fidèle Tiendrébéogo, Justin S. Pita

**Affiliations:** 1 Unité de Formation et de Recherche Biosciences, Université Félix Houphouët-Boigny (UFHB), Abidjan 22 BP 582, Côte d’Ivoire; steveseka7@gmail.com (J.S.S.S.); justin.pita@wave-center.org (J.S.P.); 2 The Central and West African Virus Epidemiology (WAVE) for Food Security Program, Pôle Scientifique et d’Innovation, Univesité Félix Houphouët-Boigny (UFHB), Bingerville 01 BP V34, Côte d’Ivoire; marius.kouassi@wave-center.org (M.K.K.); fidele.tiendrebeogo@wave-center.org (F.T.)

**Keywords:** ipomea batatas, sodium hypochlorite, mercuric chloride, genotype variability

## Abstract

Regenerating sweet potato from field-derived plant material requires careful management of several critical factors, including the effectiveness of the disinfectant, the age of the explant, and the genotype used. In this context, establishing a reliable aseptic protocol is essential for successful in vitro culture. This study aimed to assess the effects of two disinfectants (sodium hypochlorite and mercuric chloride), three sweet potato genotypes (Nakabo, Boyapleu, and Irene), and three explant ages (2, 3, and 4 weeks) on clean culture establishment and regeneration efficiency from nodal explants. The findings revealed that regeneration success is significantly influenced by the type and concentration of disinfectant, explant age, and genotype. Treatment with 10% sodium hypochlorite markedly reduced contamination, achieving clean culture and regeneration rates of 75.72 ± 3.36% and 86.83 ± 3.02%, respectively, regardless of explant age. In contrast, higher concentrations of mercuric chloride induced necrosis in the explants. The highest clean culture rate (93.82 ± 1.16%) was observed in 3-week-old explants, which also showed a regeneration rate of 54.93 ± 3.19%. Furthermore, the Boyapleu and Irene genotypes demonstrated good suitability for in vitro culture, whereas the Nakabo genotype performed poorly under the tested conditions.

## 1. Introduction

Sweet potato (*Ipomoea batatas* [L.] Lam) is a vital staple crop, particularly in tropical and subtropical regions, where it contributes significantly to food and nutritional security. Ranked as the sixth most important food crop globally after rice, wheat, maize, potato, and cassava it is a strategic resource in the fight against hunger [1,2]. However, its cultivation in sub-Saharan Africa and other parts of the world remains constrained by several challenges, notably the lack of high performing, locally adapted genotypes and widespread incidence of pests and diseases [3]. Inadequate seed systems often lead to the use of poor quality planting materials, facilitating the spread of bacterial and viral pathogens and resulting in substantial yield losses, with viral infections alone responsible for annual reductions ranging from 56% to 100% [4,5].

In this context, in vitro propagation emerges as a reliable technique for the rapid multiplication of healthy, virus-free plantlets, offering a promising solution for improving sweet potato production [6,7]. However, the success of in vitro culture is highly dependent on effective explant disinfection. The main objective of this step is to eliminate surface and internal contaminants without compromising explant viability or regenerative capacity [8,9]. Several disinfectants such as ethanol, hydrogen peroxide, bromine water, silver nitrate, calcium hypochlorite, antibiotics, mercuric chloride, and sodium hypochlorite have been tested across crops [10]. Among these, mercuric chloride and sodium hypochlorite remain the most widely used due to their broad spectrum antimicrobial activity [11]. Nevertheless, their application requires careful optimization [11,12,13]. Furthermore, factors such as the physiological age of the explant and the genotype can significantly influence both the success of disinfection and the regenerative response during in vitro culture initiation [12,13]. This finding was observed not only with the studies of Ahanhanzo et al. [14] carried out on teak plants but also by Yesmin et al. [15] on ginger culture.

Most existing studies have examined individual variables, typically the concentration of disinfectants [16,17], or at best, two-factor interactions such as disinfectant type with treatment duration [18,19], or disinfectant type with genotype [12]. However, the combined interaction among disinfectant type, explant age, and genotype remains largely unexplored.

This gap is particularly significant for nodal explants, which are crucial for direct regeneration [20] yet highly vulnerable to contamination. For instance, Onwunkibo et al. [21] reported the low effectiveness (≤40%) of NaOCl on nodal explants of sweet potato in Nigeria, without considering the donor plant’s age or genotype. Likewise, Doussoh et al. [22], in their study on three local sweet potato varieties (“Dokoun”, “Vobodouaho”, and “Djlodou”), found that a 0.5% concentration of mercuric chloride yielded the highest survival rate (83.33%). However, the outcome was strongly genotype-dependent. Hammond et al. [23] compared the effectiveness of mercuric chloride and sodium hypochlorite for microbial decontamination and found mercuric chloride (20 min exposure) to be the most effective. Yet, its phytotoxicity, especially at longer exposures, and the absence of explant age as a factor were notable limitations. Similarly, Pérez et al. [13] used a combined treatment involving povidone–iodine, 2% sodium hypochlorite, acetic acid, and quaternary ammonium compounds. While this approach reduced contamination rates to 10% and eliminated 70% of microbial presence, it also failed to consider the influence of explant age and genotype.

The lack of systematic assessment of the disinfectant–age–genotype interaction represents a critical bottleneck in developing reproducible and efficient disinfection protocols, particularly in Côte d’Ivoire, where no study has yet addressed this issue.

Given these gaps, our study presents for the first time the combined effects of disinfectant type and concentration, explant age, and genotype on the efficiency of in vitro culture establishment in sweet potato. By evaluating eight disinfection treatments using sodium hypochlorite and mercuric chloride on nodal explants of varying ages from three sweet potato genotypes, we aim to fill a critical methodological gap. Unlike previous studies that focused on isolated or dual-factor analyses, this work provides a comprehensive assessment of the three-way interaction between key variables that affect in vitro culture success. The objective was to identify the optimal conditions that minimize microbial contamination while maximizing culture cleanliness and regeneration success.

## 2. Materials and Methods

### 2.1. Plant Material

**Figure 1:**
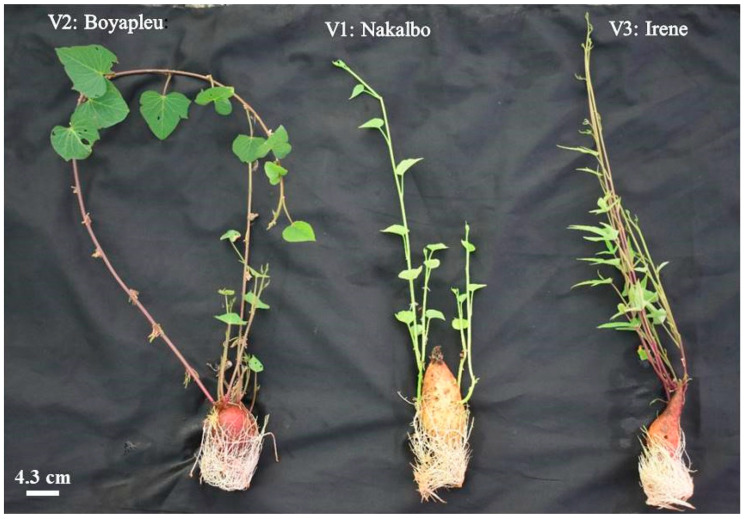
Different sweet potato varieties used.

The plant material used in this study consisted of stem fragments from three sweet potato genotypes: Boyapleu, Irene, and Nakalbo. The characteristics of these genotypes are summarized in Table 1. The Boyapleu genotype is cultivated in Côte d’Ivoire, specifically in the northwestern region, whereas the Irene and Nakalbo genotypes were provided by the National Institute for Environment and Agricultural Research (INERA) in Burkina Faso (Figure 1).

### 2.2. Preparation of Plant Material

The sweet potato tubers were harvested, washed, and treated with a mancozeb based fungicide, sold under the name ‘Ivory’ (UPL Corp. company Callivoire, Abidjan/Côte d’Ivoire). The fungicide was prepared at a concentration of 128 mg/L. The tubers were then rinsed with sterile water and air dried at room temperature for one day. After drying, the tubers were placed in 350 mL disposable cups, two-thirds filled with water, which was renewed every other day for four weeks. The plants regenerated from these tubers were used as plant material for in vitro initiation.

### 2.3. Preparation of the Culture Medium

The culture medium was prepared using the mineral components of Murashige and Skoog (Duchefa/Haarlem, The Netherlands) [24], supplemented with 1 g/L of myo-inositol (Duchefa/Haarlem, The Netherlands), 30 g/L of sucrose (Sucaf/Ferkessédougou, Côte d’Ivoire), and 0.01 mg/L of naphthaleneacetic acid (NAA) (Duchefa/Haarlem, The Netherlands). The pH of the medium was adjusted to 5.7 ± 0.1 using 1 N sodium hydroxide (NaOH) (Merck/Darmstadt, Germany). or 1 N hydrogen chloride (HCl) (Merck/Darmstadt, Germany). To solidify the medium, 7 g/L of agar was added (Duchefa/Haarlem, The Netherlands). Subsequently, 10 mL of the prepared medium was dispensed into test tubes, which were then sterilized in an autoclave at 121 °C for 20 min at 1 bar of pressure.

### 2.4. Disinfection of Plant Material

#### 2.4.1. Collection and Preparation

Nodal segments were collected from 2-, 3-, and 4-week-old plants of the three sweet potato genotypes. A total of 80 nodal segments per genotype and age group were collected. Under a laminar flow hood, the explants were stripped of their leaves, rinsed with sterile distilled water, and immersed in 70% ethanol (Aci-chimie/Abidjan, Côted’Ivoire) for five minutes. They were then treated for 20 min in either mercuric chloride (MC) (VWR-Chemicals, Bangalore, India) solutions of varying concentrations or sodium hypochlorite solutions (SH) (Sipro-Chim/Abidjan, Côted’Ivoire) containing a few drops of Tween 20 (VWR-Life science, Chicago, IL, USA) (one drop per 100 mL of solution), as detailed in Table 2. The explants were then rinsed three times with sterile distilled water before being cultured on nutrient media.

#### 2.4.2. Inoculation

**Figure 2:**
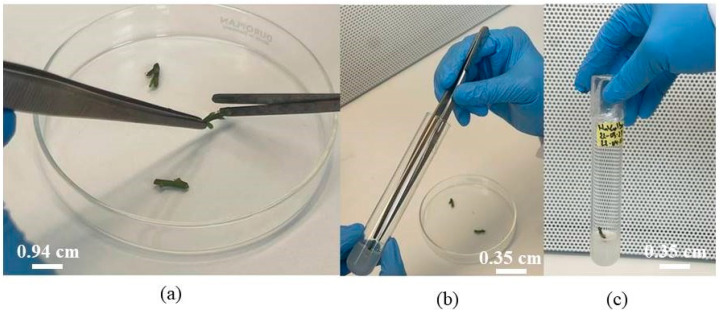
The various stages of cultivation: (**a**) collection of uni-nodal segments; (**b**) culture of uni-nodal segments in test tubes; (**c**) sealed and labeled tube with segment in in vitro culture.

Under a laminar flow hood, explants were excised and inoculated into test tubes at a rate of one explant per tube (Figure 2). The tubes were labeled and sealed, then placed in a growth chamber at 25 °C ± 1 °C with 5000 Lux light intensity and a 16 h photoperiod.

### 2.5. Evaluated Parameters

Weekly evaluations were conducted over an 8-week period to assess clean culture rates, as well as rates of regeneration and necrosis.

#### 2.5.1. Assessment of the Antiseptic Properties of Disinfectants (Clean Culture Rates: CCR)

The presence of fungal spores or a milky, pink, black, yellow, or whitish film at the interface between tissue and culture medium indicated the presence of explant associated infection. Fungal spores were also observed directly on the explants. Explants showing none of these characteristics were isolated and counted after two weeks of culture, and then again every two weeks for the eight-week period. The clean culture rate was calculated using the following formula:

(1) $$\mathrm{CCR}\left(\%\right)=\frac{\mathrm{Number}\;\mathrm{of}\;\mathrm{uninfected}\;\mathrm{explants}}{\mathrm{Total}\;\mathrm{number}\;\mathrm{of}\;\mathrm{explant}}\times 100$$

#### 2.5.2. Assessment of Regeneration Rate (RR)

The regeneration rate was determined by counting the number of leaves and/or roots produced in each tube. This was calculated from the second week onwards using the following formula:

(2) $$\mathrm{RR}\left(\%\right)=\frac{\mathrm{Number}\;\mathrm{of}\;\mathrm{explants}\;\mathrm{that}\;\mathrm{have}\;\mathrm{produced}\;\mathrm{leaves}\;\mathrm{and}\;\mathrm{or}\;\mathrm{roots}}{\mathrm{Total}\;\mathrm{number}\;\mathrm{of}\;\mathrm{explant}}\times 100$$

#### 2.5.3. Evaluation of Necrosis Rate (NR)

Necrosis in the explants was identified by tissue death, which manifested as brown or black discoloration. This was assessed through direct observation and counting of explants exhibiting these characteristics. The necrosis rate (NR) was calculated using the following formula:

(3) $$\mathrm{NR}\left(\%\right)=\frac{\mathrm{Number}\;\mathrm{of}\;\mathrm{necrotic}\;\mathrm{explant}\;\mathrm{or}\;\mathrm{plantlet}}{\mathrm{Total}\;\mathrm{number}\;\mathrm{of}\;\mathrm{explant}}\times 100$$

### 2.6. Statistical Analysis

The collected data were analyzed using R software version 4.2.1. The non-parametric Kruskal–Wallis test was used to analyze the effects of one way such as disinfecting agents at their different concentrations (8 treatments), sweet potato genotypes (3 genotypes), and explant age (3 types). When significant differences were observed, pairwise comparisons were conducted using the Wilcoxon test at a 5% significance level. Regarding their interactions, we used a generalized linear model (GLM) with a Poisson distribution for a bidirectional effect. When significant differences were observed between means, we used the emmeans library to estimate adjusted marginal means and pairwise comparisons with Tukey’s *p*-value adjustment to separate means with the theoretical statistics value α = 0.05.

## 3. Results

### 3.1. Appearance and Color of Contamination Observed in Cultures After Disinfection

**Figure 3:**
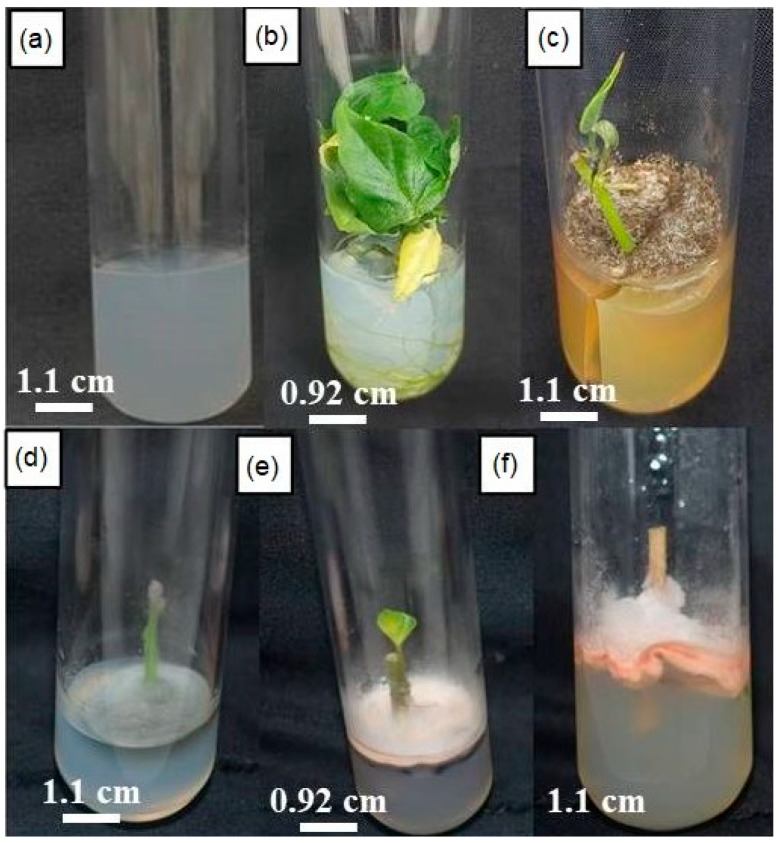
Different types of in vitro cultures: (**a**) uncontaminated control medium; (**b**) uncontaminated plant; (**c**) white-pink contaminated medium; (**d**) pink contaminated medium; (**e**) gray contaminated medium; (**f**) brown contaminated medium. The evaluation was carried out after 4 weeks of cultivation.

Figure 3a shows an uncontaminated explant free medium acting as a control. Despite the use of disinfectants such as mercuric chloride and sodium hypochlorite, some cultures were found to be contaminated. These contaminations were characterized by significant variations, not only in texture, ranging from translucent to intensely pigmented, and sometimes presenting a frothy appearance characteristic of specific fungi. Figure 3 below shows the contaminations obtained. While some explants remained free of infection (Figure 3b), others showed a whitish, powdery contamination, easily identifiable by a thin white veil around the explant (Figure 3e). In addition, some contaminations were yellow-green with a powdery texture at the base of the plant, while others were distinguished by a dark gray to black coloration, also powdery (Figure 3d). Finally, cases of mixed contamination were observed, including superimpositions of pink-orange and white mycelium (Figure 3e,f).

### 3.2. Influence of Sodium Hypochlorite and Mercury Chloride Concentrations on the Clean Culture and Regeneration Rates of Explants

**Figure 4:**
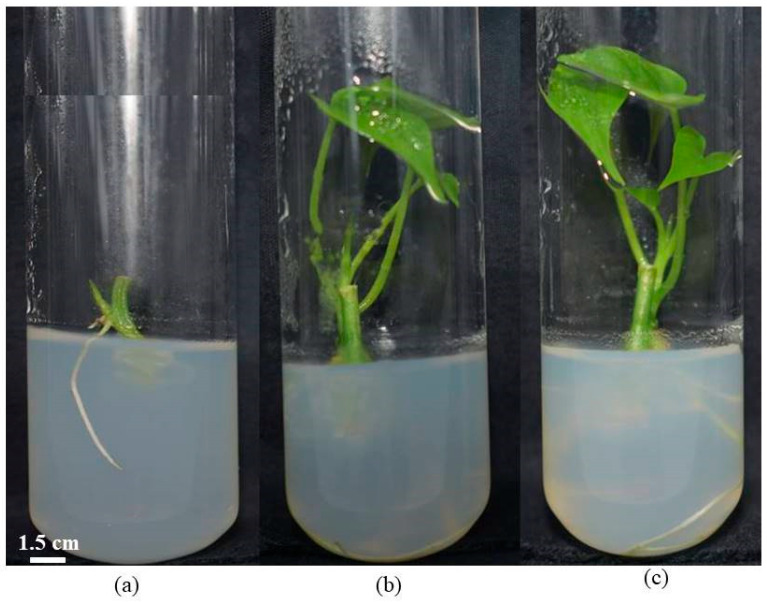
Different types of regeneration observed: (**a**) regeneration by emission of preleaf and root; (**b**) regeneration by emission of leafy stem only; (**c**) regeneration by leafy stem and root.

Table 3 presents the effects of different disinfectant treatments on the percentage of clean cultures and the regeneration capacity of sweet potato explants. Statistically high levels of clean culture were recorded with all mercuric chloride treatments, exceeding 91%, particularly at concentrations of 0.05%, 0.1%, 0.2%, and 0.3%. In contrast, sodium hypochlorite treatments resulted in lower clean culture rates, ranging from 67.48 ± 3.92% to 75.72 ± 3.36%, with the lowest value observed in SH4 (75.72 ± 3.36%). Regarding regeneration, the treatments had variable effects. Explant regeneration was evidenced by the emergence of leaflets (Figure 4), and a general decline in regeneration percentage was observed with increasing disinfectant concentrations. Sodium hypochlorite treatments were more favorable to regeneration compared to mercuric chloride treatments (Table 3). The highest regeneration rate was recorded with 10% sodium hypochlorite (86.83 ± 3.02%), followed by 30% sodium hypochlorite (77.36 ± 4.04%). In contrast, the lowest regeneration rates were observed with 0.2% and 0.3% mercuric chloride treatments (1.23 ± 0.70%).

### 3.3. Effect of Explant Age on Clean Culture and Regeneration Rates

**Figure 5:**
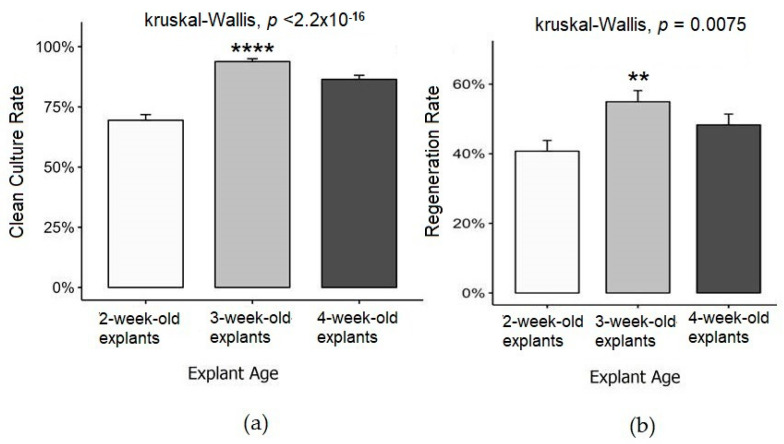
Influence of explant age on clean culture rate and regeneration rate: (**a**) effect of explant age on clean culture rate; (**b**) effect of explant age on regeneration rate. Statistical significance was determined using the Kruskal–Wallis test (clean culture rate: **** *p* < 0.0001; regeneration rate: ** *p* < 0.01).

The explants from 3-week-old plants exhibited the highest clean culture rate (93.82 ± 1.16%), followed by those from 4-week-old plants (86.41 ± 1.69%). Conversely, 2-week-old explants were the most contaminated, with a significantly lower clean culture rate of 69.44 ± 2.33% (Figure 5a). The highest regeneration rate was observed in 3-week-old explants (54.93 ± 3.19), followed by 4-week-old explants (48.30 ± 3.09%) and 2-week-old explants (40.74 ± 3.08%) (Figure 5b). Three-week-old explants demonstrated the best overall performance, achieving high regeneration rates alongside a relatively low contamination rate. 

### 3.4. Effect of Genotypes on Clean Culture and Regeneration Rates

**Figure 6:**
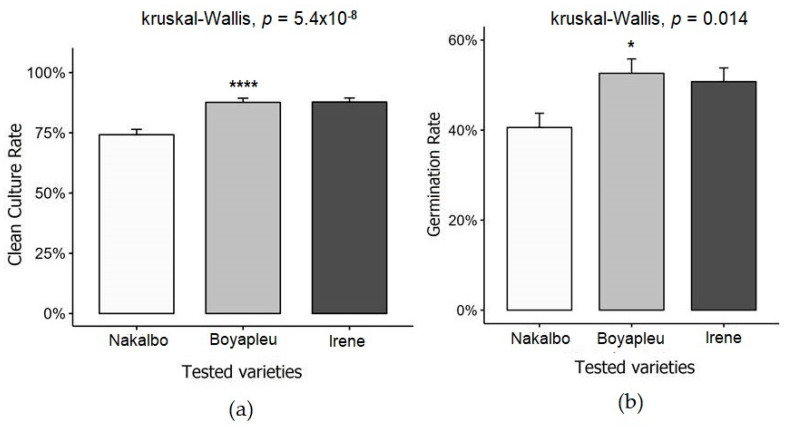
Changes in variety clean crop rates over time: (**a**) effect of varieties on clean culture rate; (**b**) effect of varieties on regeneration rate; Statistical significance was determined using the Kruskal-Wallis test (self-cultivation rate: **** *p* < 0.0001; regeneration rate: * *p* < 0.05).

Figure 6 shows the clean culture and regeneration rates of three sweet potato genotypes: Nakalbo, Boyapleu, and Irene. The results indicate a highly significant effect of genotype on the clean culture rates. Nakalbo displayed the lowest clean culture rate at 74.23% (Figure 6a). By contrast, Boyapleu and Irene demonstrated considerably higher clean culture rates of 87.65% and 87.80%, respectively. Regarding the regeneration rate, varietal influence was less pronounced. Nakalbo exhibited the lowest regeneration rate at 46.40%, while Boyapleu and Irene showed higher regeneration rates of 52.62% and 50.77%, respectively.

### 3.5. Simultaneous Influence of Genotypes and Treatments on the In Vitro Clean Culture and Regeneration Rates of Explants

The SH3 treatments (30% sodium hypochlorite) was the most effective for the Boyapleu genotype, achieving a clean culture and regeneration rates of 85.18 ± 3.16% and 83.95 ± 4.42%, respectively. For the Irene genotype, SH3 recorded the highest regeneration rate (91.36 ± 2.97%) and a clean culture rate of 70.37 ± 4.76%. For Nakalbo, although SH3 achieved moderate regeneration (56.79 ± 5.87%) and clean culture (71.60 ± 3.91%) rates, which were inferior to those for Boyapleu. All sodium hypochlorite treatments yielded clean culture rates exceeding 51.85 ± 5.53%, with regeneration rates ranging from 34.57 ± 5.83% to 92.59 ± 2.90%, irrespective of genotype. The Irene and Boyapleu genotypes performed better, showing regeneration and clean culture rates above 70% for all sodium hypochlorite treatments, in contrast to the Nakalbo genotype. Regarding mercuric chloride treatments, only MC1 (0.05% concentration) supported regeneration across all genotypes. MC2 achieved clean culture rates above 70%, but regeneration was limited to the Irene and Boyapleu genotypes, with very low rates below 18%. Treatments MC3 and MC4 showed no regeneration (0%) for any genotype, rendering them ineffective for explant regeneration. In summary, SH3 is the optimal treatment, offering a balance of high regeneration rates and moderate clean culture rates (over 56%) for all genotypes (Table 4).

### 3.6. Simultaneous Influence of Explant Age and Treatments on Clean Culture and Regeneration Rates In Vitro

Table 5 shows the simultaneous effect of explant age and different treatments on the rate of clean culture and the in vitro regeneration of sweet potato seedlings. For 2-week-old explants, T3 (30% sodium hypochlorite) exhibited a high regeneration rate (85.19 ± 4.23%) comparable to that of T1 (86.42 ± 3.82%) while maintaining a relatively high clean culture rate (54.32 ± 3.80%). The highest clean culture rates for this age group were achieved with mercuric chloride treatments (exceeding 70%); however, these treatments recorded the lowest regeneration rates (MC1 = 34.57 ± 5.55%) or no regeneration at all (MC2, MC3, and MC4 = 0%).

For 3-week-old explants, all sodium hypochlorite treatments and MC1 (mercuric chloride) demonstrated high regeneration rates exceeding 70%, as well as significant clean culture rates, surpassing 80%. In contrast, treatments MC3, MC3, and MC4 achieved clean culture rates above 80% but displayed extremely low regeneration rates of below 4%. In 4-week-old explants, regeneration was moderate. Treatment SH1 showed the best performance (80.25 ± 4.40%) and a clean culture rate of 80.24 ± 3.61%, followed by treatments MC1 and SH4. Treatments MC2 and MC3 yielded almost no regeneration, despite achieving clean culture rates approaching 100%. Three-week-old explants offered the best balance between high regeneration and clean culture rates, particularly with treatments SH2 and SH3. Two-week-old explants exhibited high regeneration but often at the expense of clean culture rates for some treatments. While potentially detrimental for 2-week-old explants and 3-week-old explants, treatments MC1 and MC2, based on mercuric chloride, achieved regeneration of above 30% in 4-week-old explants.

### 3.7. Effect of Different Treatments on Necrosis Rate

**Figure 7:**
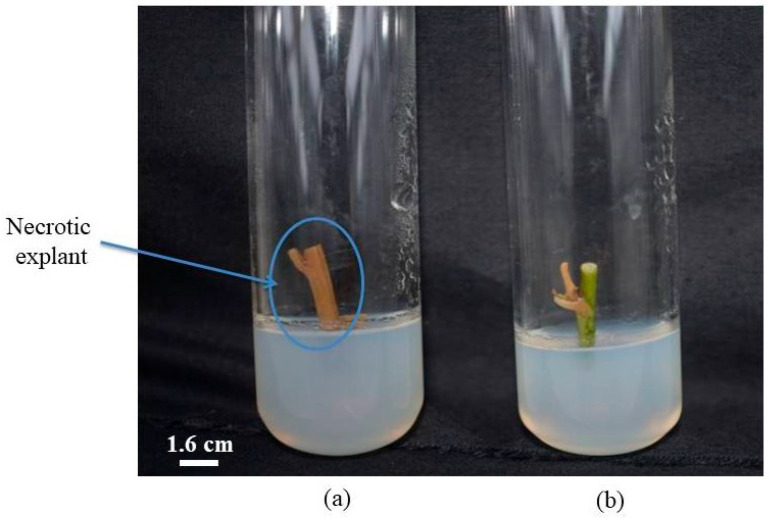
Comparative illustration of a necrotic and a healthy explant after disinfection: (**a**) necrotic explant; (**b**) healthy explant (image taken after the first week of cultivation).

**Figure 8:**
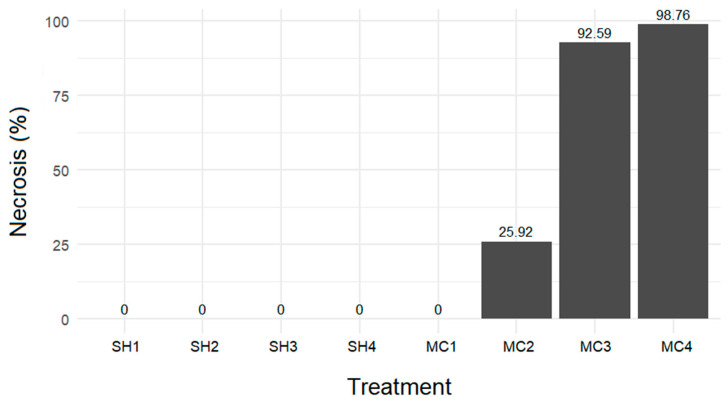
Necrosis rate as a function of the different treatments performed Concentrated hypochlorite solution at the following values: SH1 = 10%; SH2:20%; SH3: 30%; and SH4: 40%. Concentrated mercuric chloride solution: MC1 = 0.05%; MC2: 0.1%; MC3: 0.2%; and MC4: 0.3%. The explants were subjected to a 20 min treatment.

The necrosis rate varied depending on the treatment applied. Only explants that turned brown or black were considered necrotic (Figure 7). Figure 8 illustrates the necrosis rates associated with the different treatments. Treatments with 10%, 20%, 30%, and 40% concentrated sodium hypochlorite and 0.05% concentrated mercuric chloride did not result in necrosis. In contrast, treatments with 0.1%, 0.2, and 0.3% concentrated mercuric chloride resulted in necrosis rates of 25.92%, 92.59%, and 98.76%, respectively.

## 4. Discussion

The in vitro culture of sweet potato (*Ipomoea batatas*) represents an essential strategy for the production of pathogen-free planting material, particularly in regions facing high viral pressure and limited access to healthy cuttings [25,26]. However, the successful establishment of cultures from field-collected material remains constrained by several critical factors [19,27]. Among these, the efficacy of the disinfectant, the physiological age of the explants, and the genetic variability of the genotypes play a determining role in microbial contamination, tissue survival, and in vitro regenerative capacity [28,29]. Although these factors have been individually studied in the literature, their combined interactions have rarely been explored in an integrated manner. In the present study, a multifactorial approach was adopted to assess the cross-effects of disinfectant type (sodium hypochlorite vs. mercuric chloride), explant age, and genotype on the quality of in vitro disinfection.

The results revealed significant differences in clean culture rate, fungal contamination, tissue necrosis, and regeneration capacity. Sodium hypochlorite, particularly at certain concentrations, yielded the highest clean culture rates (up to 73.35 ± 1.79%) and regeneration rates (77.26 ± 1.97%), while preventing tissue necrosis. In contrast, although mercuric chloride was effective in reducing contamination, it exhibited phytotoxic effects at higher concentrations, severely limiting explant regeneration (18.72 ± 1.99%). These findings confirm previous observations regarding the deleterious effects of high disinfectant doses, which can cause cellular damage [23], as well as the proven efficacy of sodium hypochlorite in reducing microbial contamination in plant tissue cultures [30,31].

Explant age also significantly influenced the outcomes. Very young explants (from two-week-old plants) were more susceptible to contamination, exhibiting high contamination rates (30.55 ± 2.33%) regardless of the treatment applied. This observation aligns with the work of Ponce de León and Montesano [32], who suggested that immature tissues are more vulnerable to pathogens due to their underdeveloped metabolism and incomplete defense systems. In contrast, explants from three-week-old plants showed greater tolerance, likely due to more stable cell structures and increased resistance to chemical stress. These findings also corroborate those of Ahanhanzo et al. [14], who noted that the efficacy of disinfectants is influenced by the structural characteristics of plant tissues, including their capacity to absorb or resist chemical agents.

Moreover, genotypic variability strongly influenced the results. The Nakalbo genotype exhibited the lowest clean culture rate (74.23%), whereas the Boyapleu and Irene genotypes performed better, with clean culture rates of 87.65% and 87.80%, respectively, indicating greater resilience to microbial and chemical stress. This result confirms the importance of genotype in explant responses to disinfection protocols, as also emphasized by Santos and Olivares [29] in their study on genotype-dependent modulation of in vitro performance in sweet potato. Similar observations were reported by Dossouh et al. [12] in a study on the in vitro disinfection of three local sweet potato varieties from Benin.

Fungal contamination emerged as the primary challenge during the in vitro introduction phase. The absence of contamination in control media without explants (Figure 3a) confirmed the effectiveness of the autoclaving parameters (temperature, pressure, duration) and validated the rigor of the sterilization process. Nevertheless, despite the strict application of disinfection protocols, a wide range of contaminant types was observed, reflecting the limitations of certain chemical treatments [30,31].

Fungal contaminants were attributed to several pathogenic genera, based on visual characteristics (color, texture, sporulation) described by Jazmín Pérez-Pazos et al. [13]. These included *Sarocladium subulatum* (whitish mycelium), *Aspergillus* sp. (yellow-green deposits), Cladosporium sp. (dark, sporulating structures), and *Fusarium* sp. Mixed infections were also detected, highlighting the complexity of microbial communities associated with field-derived explants. These results are consistent with the findings of Jena and Samal [16] and underscore the necessity of adopting rigorous aseptic techniques throughout all stages of the process [23,33].

## 5. Conclusions

This study showed that improving in vitro sweet potato regeneration requires controlling explant age and disinfection, as well as taking into account variability in genotypic response. Our results indicate that while mercuric chloride was more effective in reducing contamination, it also induced tissue necrosis. In contrast, sodium hypochlorite supported better explant viability and promoted regeneration. The treatment with 10% sodium hypochlorite for 20 min proved effective in striking a balance between reducing contamination and promoting regeneration across all explant types, making it a suitable and adaptable option for sweet potato tissue culture. Additionally, explants from three-week-old plants consistently exhibited lower contamination rates, confirming this age as optimal for in vitro introduction. Genotypic variation also influenced regeneration, likely due to inherent genetic traits or differential responses to culture conditions. In this study, the Boyapleu and Irene genotypes were less susceptible to contamination. These findings provide valuable insights for refining in vitro culture protocols, thereby advancing plant biotechnology and contributing to improved agricultural productivity.

This study proposes an integrated methodological framework based on three key advances: (1) the identification of 10% sodium hypochlorite (applied for 20 min) as an effective and less phytotoxic alternative to mercuric chloride, capable of achieving both efficient decontamination and successful regeneration; (2) the establishment of three-week-old explants as the optimal age for minimizing initial microbial load; and (3) the identification of genotypes such as Boyapleu and Irene as particularly well-suited for in vitro culture, underscoring the impact of genetic variability on protocol outcomes. This integrative strategy lays the foundation for a reproducible and adaptable micropropagation protocol for sweet potatoes, filling the methodological gaps left by previous studies focused on isolated parameters.

## Figures and Tables

**Table 1:** Characteristics of the sweet potato (*Ipomoea batatas* [L.] Lam) genotypes used.

Characters	Genotypes		
	Nakalbo	Boyapleu	Irene
Origin	Côte d’Ivoire	Burkina Faso	Burkina Faso
Plant type	Spreading	Spreading	Erect
General leaf outline	Cordate	Cordate	Hastate
Leaf lobe number	1	3	3
Skin color	Cream	purple-red	purple-red
Flesh color	White	White	Orange

**Table 2:** Different concentrations of sodium hypochlorite and mercury chloride used for 20 min.

Type of Agent Disinfecting	Concentration (%)	Treatments Codes
Sodium hypochlorite (NaOCl)	10.00	SH1
	20.00	SH2
	30.00	SH3
	40.00	SH4
Mercuric chloride (HgCl_2_)	0.05	MC1
	0.10	MC2
	0.20	MC3
	0.30	MC4

**Table 3:** Effect of different treatments on clean culture rate and regeneration of sweet potato used for 20 min.

Treatments	Clean Culture Rates (%)	Regeneration Rates (%)
10% of NaOCl	75.72± 3.36 b	86.83± 3.02 a
20% of NaOCl	67.48 ± 3.92 c	78.18 ± 3.56 ab
30% of NaOCl	75.72 ± 3.31 b	77.36 ± 4.04 b
40% of NaOCl	74.48 ±3.71 bc	66.66 ± 4.72 c
0.05% of HgCl_2_	91.58 ± 3.5 a	62.13 ± 4.78 c
0.1% of HgCl_2_	93.82 ± 1.95 a	10.28 ± 2.90 d
0.2% of HgCl_2_	94.23 ± 1.92 a	1.23 ± 0.70 d
0.3% of HgCl_2_	93.00 ± 2.16 a	1.23 ± 0.70 d
*p*	<0.0001	<0.0001

NaOCl: Sodium hypochlorite; HgCl_2_: mercuric chloride (HgCl_2_). Treatments were carried out for a duration of 20 min. For each parameter per row, the values (mean ± standard error) with different letters are not significantly different between type of agent disinfecting and treatments according to non-parametric Kruskal–Wallis and pairwise Wilcoxon tests (*p* < 0.05).

**Table 4:** Effect of genotype and treatments on the clean culture and in vitro regeneration rates of explants of sweet potato.

Genotypes	Treatments	Clean Culture Rates (%)	Regeneration Rate (%)
Nakalbo	10% of NaOCl	64.19 ± 4.15 h	92.59 ± 2.90 l
	20% of NaOCl	51.85 ± 5.53 i	77.78 ± 4.87 m
	30% of NaOCl	71.60 ± 3.91 g	56.79 ± 5.87 jk
	40% of NaOCl	62.96 ± 4.59 h	34.57 ± 5.83 l
	0.05% of HgCl_2_	87.65 ± 3.59 cd	62.96 ± 6.33 de
	0.1% of HgCl_2_	86.41 ± 3.38 cde	0.00 ± 0.00 ef
	0.2% of HgCl_2_	88.88 ± 3.55 c	0.00 ± 0.00 d
	0.3% of HgCl_2_	80.24 ± 4.02 f	0.00 ± 0.00 h
Boyapleu	10% of NaOCl	79.01 ± 4.00 f	81.48 ± 4.23 hi
	20% of NaOCl	72.83 ± 3.98 g	83.95 ± 4.42 j
	30% of NaOCl	85.18 ± 3.16 de	83.95 ± 4.42 fg
	40% of NaOCl	71.60 ± 5.58 g	86.42 ± 3.82 jk
	0.05% of HgCl_2_	98.76 ± 0.87 ab	66.67 ± 5.62 ab
	0.1% of HgCl_2_	97.53 ± 1.74 ab	18.52 ± 4.92 bc
	0.2% of HgCl_2_	97.53 ± 1.21 ab	0.00 ± 0.00 bc
	0.3% of HgCl_2_	98.76 ± 0.87 ab	0.00 ± 0.00 ab
Irene	10% of NaOCl	83.95 ± 3.85 e	86.42 ± 3.82 g
	20% of NaOCl	77.77 ± 4.17 f	72.84 ± 3.78 i
	30% of NaOCl	70.37 ± 4.76 g	91.36 ± 2.97 k
	40% of NaOCl	88.88 ± 2.17 c	79.01 ± 4.89 d
	0.05% of HgCl_2_	87.65 ± 3.36 cd	56.79 ± 5.73 de
	0.1% of HgCl_2_	97.53 ± 1.21 ab	12.35 ± 3.36 bc
	0.2% of HgCl_2_	97.53 ± 1.45 ab	3.70 ± 1.45 c
	0.3% of HgCl_2_	100.00 ± 0.00 a	3.70 ± 1.45 a
*p*		<0.0001	<0.0001

NaOCl: Sodium hypochlorite; HgCl_2_: mercuric chloride (HgCl_2_). Treatments were carried out for a duration of 20 min. For each parameter per row, the values (mean ± standard error) with different letters are not significantly different between sweet potato genotype and treatments according to non-parametric Kruskal–Wallis and pairwise Wilcoxon tests (*p* < 0.05).

**Table 5:** Influence of explant ages and disinfection treatments on clean cultures and in vitro explant regeneration of sweet potato.

Age of explants	Treatments	Clean Culture Rates (%)	Regeneration Rate (%)
4-week-old explants	10% of NaOCl	80.24 ± 3.61 h	80.25 ± 4.40 e
	20% of NaOCl	62.96 ± 4.59 k	66.67 ± 5.48 g
	30% of NaOCl	74.07 ± 4.04 j	66.67 ± 5.33 g
	40% of NaOCl	85.18 ± 3.40 g	70.37 ± 5.38 f
	0.05% of HgCl_2_	100.00 ± 0.00 a	69.14 ± 6.28 f
	0.1% of HgCl_2_	100.00 ± 0.00 a	30.86 ± 5.17 j
	0.2% of HgCl_2_	98.76 ± 0.87 ab	0.00 ± 0.00 m
	0.3% of HgCl_2_	90.12 ± 2.76 ef	2.47 ± 1.21 kl
3-week-old explants	10% of NaOCl	92.59 ± 2.92 d	93.83 ± 2.52 b
	20% of NaOCl	91.35 ± 2.97 de	98.77 ± 0.87 a
	30% of NaOCl	98.76 ± 0.87 ab	80.25 ± 5.07 e
	40% of NaOCl	86.41 ± 3.38 g	79.01 ± 4.73 e
	0.05% of HgCl_2_	97.53 ± 1.21 b	82.72 ± 3.41 d
	0.1% of HgCl_2_	86.41 ± 3.38 g	0.00 ± 0.00 m
	0.2% of HgCl_2_	97.53 ± 1.21 b	3.70 ± 1.45 k
	0.3% of HgCl_2_	100.00 ± 0.00 a	1.23 ± 0.87 lm
2-week-old explants	10% of NaOCl	54.32 ± 4.38 l	86.42 ± 3.82 c
	20% of NaOCl	48.14 ± 4.76 n	69.14 ± 3.96 f
	30% of NaOCl	54.32 ± 3.80 l	85.19 ± 4.23 c
	40% of NaOCl	51.85 ± 5.08 m	50.62 ± 6.56 h
	0.05% of HgCl_2_	76.54 ± 4.32 i	34.57 ± 5.55 i
	0.1% of HgCl_2_	95.06 ± 2.06 c	0.00 ± 0.00 m
	0.2% of HgCl_2_	86.41 ± 3.61 g	0.00 ± 0.00 m
	0.3% of HgCl_2_	88.88 ± 3.55 f	0.00 ± 0.00 m
*p*		<0.0001	<0.0001

NaOCl: Sodium hypochlorite; HgCl_2_: mercuric chloride (HgCl_2_). Treatments were carried out for a duration of 20 min. For each parameter per row, the values (mean ± standard error) with different letters are not significantly different between sweet potato explant age and treatments according to non-parametric Kruskal–Wallis and pairwise Wilcoxon tests (*p* < 0.05).

## Data Availability

The original contributions presented in this study are included in this article. Further inquiries can be directed to the corresponding authors.
